# Are Proton Pump Inhibitors Contributing in Emerging New Hypertensive Population?

**DOI:** 10.3390/ph16101387

**Published:** 2023-09-30

**Authors:** Rohit Tayal, Sabina Yasmin, Samrat Chauhan, Thakur Gurjeet Singh, Monika Saini, Eman Shorog, Maryam M. Althubyani, Baiaan H. Alsaadi, Fatimah Aljohani, Maram A. Alenazi, Sarah A. Abutaily, Mohammad Yousuf Ansari

**Affiliations:** 1 Chitkara College of Pharmacy, Chitkara University, Rajpura 140401, Punjab, India; rohittayal1999@gmail.com (R.T.); gurjeet.singh@chitkara.edu.sa (T.G.S.); 2 Department of Pharmaceutical Chemistry, College of Pharmacy, King Khalid University, Abha 61421, Saudi Arabia; sahussain@kku.edu.sa; 3 M.M. College of Pharmacy, Maharishi Markandeshwar (Deemed to be) University, Mullana, Ambala 133207, Haryana, India; monika210692@gmail.com; 4 Swami Vivekanand College of Pharmacy, Ramnagar, Banur 140601, Punjab, India; 5 Clinical Pharmacy Department, College of Pharmacy, King Khalid University, Abha 61421, Saudi Arabia; eshorog@kku.edu.sa; 6 Department of Clinical Services, Pharmaceutical Care Services, King Salman Medical City, Ministry of Health MOH, Al Madinah Al Munawwarah 11176, Saudi Arabia; ph.maryam91@gmail.com (M.M.A.); baiaana@moh.gov.sa (B.H.A.); 7 Prince Sultan Armed Forces Hospital, Al Madenah Al Monwarah 42375, Saudi Arabia; fa.aljohani@psafhm.med.sa; 8 Pharmaceutical Care Services, King Salman Specialist Hospital, Ministry of Health (MOH), Hail 55471, Saudi Arabia; malenazi31@moh.gov.sa; 9 Ambulatory Care Clinical, Prince Sultan Military Medical City, Riyadh 12233, Saudi Arabia; sarahabutaily@hotmail.com

**Keywords:** hypertension, Proton Pump Inhibitor, adverse effects, endothelial dysfunction, hypocalcemia

## Abstract

Balancing the therapeutic advantages of a medicine with its possible risks and side effects is an important part of medical practice and drug regulation. When a drug is designed to treat a particular disease or medical condition ends up causing additional risks or side effects that lead to the development of other serious health problems, it can have detrimental consequences for patients. This article explores the correlation between persistent proton pump inhibitor (PPI) use and hypertension, a common cardiovascular ailment. While PPIs are beneficial in treating various gastrointestinal problems, their availability without a prescription has resulted in self-medication and long-term use without medical monitoring. Recent findings have revealed a link between long-term PPI usage and increased cardiovascular risks, particularly hypertension. This study investigates the intricate mechanisms underlying PPI’s effects, focusing on potential pathways contributing to hypertension, such as endothelial dysfunction, disruption of nitric oxide bioavailability, vitamin B deficiency, hypocalcemia, and hypomagnesemia. The discussion explains how long-term PPI use can disrupt normal endothelial function, vascular control, and mineral balance, eventually leading to hypertension. The article emphasizes the significance of using PPIs with caution and ongoing research to better understand the implications of these medications on cardiovascular health.

## 1. Introduction

Proton pump inhibitors (PPIs) are highly regarded for their effectiveness in addressing various stomach acid-related issues and hold a significant place in today’s medical landscape [1]. PPIs are primarily prescribed by healthcare professionals, especially doctors or gastroenterologists who specialize in treating gastrointestinal conditions. These medical professionals are trained to diagnose and manage conditions including peptic ulcers, gastroesophageal reflux disease (GERD), Zollinger-Ellison syndrome, and H. pylori infection for which PPIs are commonly prescribed [2]. PPIs act by inhibiting the stomach acid production widely used in short-term treatment of peptic ulcer disease; their use has expanded to long-term and even lifelong use in specific patient populations. This includes patients with GERD experiencing typical or atypical symptoms, as well as individuals who use aspirin or nonsteroidal anti-inflammatory drugs (NSAIDs) and are susceptible to gastrointestinal problems like perforation, bleeding, or gastric outlet obstruction [3,4]. Patients on dual antiplatelet therapy following coronary interventions are also commonly given PPIs to prevent GI bleeding [5]. PPIs are now available over the counter in numerous countries, enabling individuals to self-initiate their use without a prescription. This easy accessibility has led to an increase in self-medication and prolonged use of PPIs without a doctor’s recommendation [1]. The shift towards long-term PPI use in these cases is based on the benefits of acid suppression in managing symptoms, promoting ulcer healing, and reducing the risk of complications [6]. Recent research has shed light on a potential connection between prolonged PPI use and increased cardiovascular risks, specifically hypertension [7]. Hypertension is a prevalent and potentially serious medical condition characterized by elevated pressure in the arteries, posing risks to cardiovascular health and overall well-being. Since the etiology of hypertension is complex and multifaceted, it is very difficult to predict the exact cause of hypertension [8,9,10]. It has been noted that certain medications and treatments can occasionally lead to an increase in blood pressure, which may be termed drug-induced hypertension [11]. An increase in blood pressure due to drug-induced hypertension can stem from multiple mechanisms, with significant factors being the retention of sodium and fluids, the stimulation of the renin-angiotensin-aldosterone system (RAAS), changes in vascular constriction, or a fusion of these pathways [12]. The mechanism behind PPI-induced effects has received limited research attention, leaving an opportunity to investigate its precise cause. Preclinical studies have the potential to establish a connection of PPI long-term usage with endothelial dysfunction and Vitamin B12 deficiency. Additionally, PPIs have been associated with hypomagnesemia and hypocalcemia, which could potentially contribute to hypertension pathology. The focus of this article is to look into the complex mechanism of PPIs, investigate the underlying components that contribute to hypertension, and evaluate evidence that suggests a link between chronic usage and high blood pressure, along with the potential consequences of such an association. 

## 2. Proton Pump Inhibitor and Overuse

PPIs remain among the most frequently prescribed drugs in numerous Western nations, primarily because of their effectiveness and favorable safety record. It is undeniable that the utilization of PPIs is consistently increasing each year in nearly all developed nations and has become a concern for practitioners, as shown in Table 1 [13]. The primary causes for the excessive use of PPIs are based on the following factors: the use of PPIs for preventing ulcers in patients at low risk, the unnecessary use of PPIs in non-intensive care units for stress ulcer prophylaxis, the use of steroid therapy alone, the administration of anticoagulant treatment alone in the absence of predisposing factors for stomach and duodenal damage, excessive treatment for functional dyspepsia (FD), and an inappropriate identification of acid-related disorders [13,14,15]. In addition, the cases of these inadequate treatments are documented by general practitioners without periodic reevaluation of critical aspects of prolonged therapy [16]. The data from the study reveals that, on average, more than 57% of patients admitted to general medical wards and approximately 50% of patients treated in primary care settings are using these drugs inappropriately [17]. The increasing availability of these drugs has raised concerns about their potential impact on cardiovascular health, particularly their potential association with hypertension, which is a widespread global health problem.

## 3. PPIs over Time: Risks of Extended Use and High Dosages

**Figure 1:**
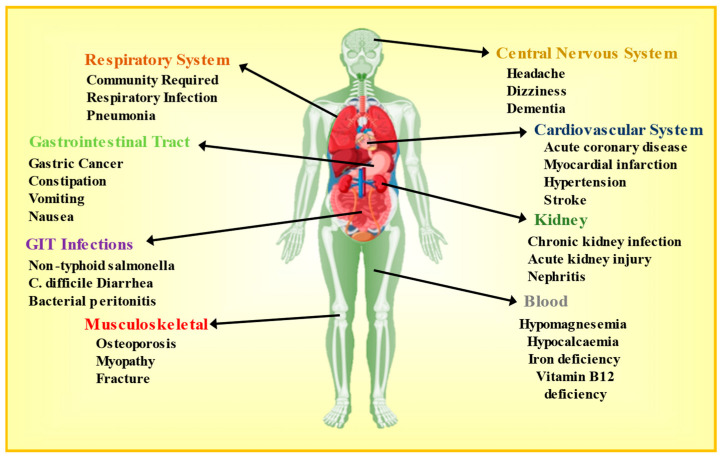
Unintended adverse effects of PPI utilization.

While it is true that PPIs are generally regarded as safe and efficiently tolerated, it is essential to note that they can still have potential adverse effects, especially with long-term or high-dose use, as shown in Figure 1. Patients may occasionally experience mild adverse effects such as rashes, dizziness, headaches, and digestive issues like constipation, nausea, stomach discomfort, flatulence, or loose stools with short-term use of PPIs, which are reversible in nature or disappear once the drug administration has been discontinued [27]. Numerous recent studies have documented that excessive prescribing of PPIs can result in a rise in the occurrence of negative outcomes among patients. These adverse events encompass enteric infections like community-acquired pneumonia, Clostridium difficile diarrhea, hip fractures, gastric carcinoids, hypomagnesemia, nutritional deficiencies, as well as chronic kidney disease, ischemic heart disease, and dementia [28]. Additionally, it is important to consider the potential serious and long-term effects associated with PPI use. While PPIs are typically well-accepted, there have been reports of more significant adverse effects that should be taken into consideration. Long-term PPI use has been connected with certain risks, including an increased likelihood of bone fractures, especially among elderly individuals or those with other risk factors for osteoporosis [29]. Prolonged use of PPIs may also lead to vitamin and mineral deficiencies, such as magnesium, calcium, and vitamin B12, especially in elderly and malnourished patients, which can have implications for overall health [30]. There have been suggestions of potential links between extended usage of PPIs and an increased risk of certain conditions, like gastric cancer, chronic kidney disease, gastrointestinal infection, loss of memory, respiratory infection, and community-required pneumonia [27,31]. The utilization of PPIs has been independently associated with an increased risk of experiencing an initial cardiovascular incident. This association aligns with existing findings suggesting that PPIs can have negative effects on vascular function [32]. One of the case–control studies demonstrated that long-term usage of PPIs at a low dosage was found to be significantly linked to a 29% increased absolute risk of experiencing an ischemic stroke as well as a 36% increased absolute risk of having a myocardial infarction (MI) when compared to those not undergoing prolonged PPI treatment [33]. Another study on clinical trial data of patients solely using PPIs discovered a significant link between intake of PPIs and a 70% higher risk of experiencing cardiovascular events in individuals with GERD. Particularly, omeprazole, among the various PPIs, exhibited a stronger affinity for elevating cardiovascular risks. Additionally, prolonged PPI therapy demonstrated an augmented likelihood of adverse cardiovascular events [34]. However, various studies indicated that PPI induced cardiovascular effects along with a possible pathway, but the relationship between hypertension and chronic use of PPI is still under research as hypertension is multifactorial and finding out the exact etiology behind this is quite difficult [35]. According to the WHO, approximately 1.28 billion individuals aged 30 to 79 globally are affected by hypertension, with the majority (around 70%) living in nations with lower to middle-income levels. Recently, a pharmacovigilance investigation was carried out to investigate the link between PPI usage and the occurrence of cardiovascular events using data obtained from the database of the FDA adverse event reporting system spanning the period of 2015 to 2019. The findings of this study indicated that the impact on vascular function outweighed the effect on cardiac function, suggesting a more substantial influence on blood vessels [35]. 

## 4. Impacting Blood Pressure: PPIs and Their Potential Association with Hypertension

Although chronic intake of PPIs has an indirect association with hypertension, there are some complex pathways that may initiate after chronic intake of PPIs and may ultimately be responsible for hypertension. Following are the possible pathways that can initiate the cascade of a rise in blood pressure when abusing PPIs or properly used for the long-term.

### 4.1. PPIs and Their Influence on Dimethylarginine Dimethylaminohydrolase Enzyme: Implications for Endothelial Dysfunction

**Figure 2:**
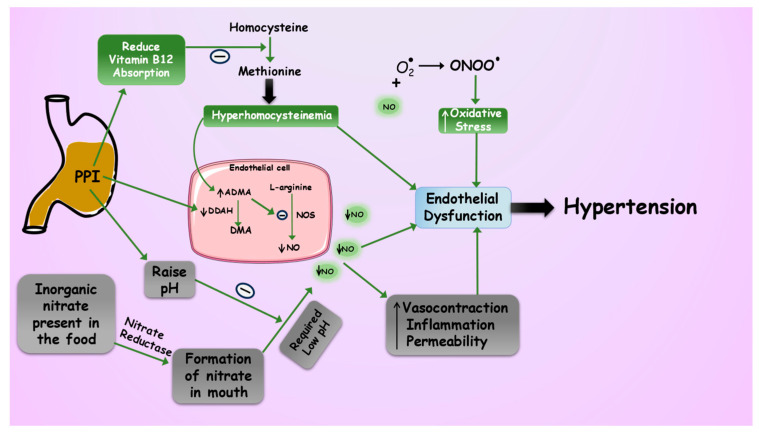
A Possible pathway connecting chronic PPI utilization with hypertension involves several mechanisms. This pathology of hypertension commences with PPIs impeding the activity of the DDAH enzyme responsible for ADMA metabolism. This heightened ADMA level reduces NO synthesis and induces vasoconstriction. Furthermore, PPIs hinder the conversion of inorganic nitrate in food to plasma NO by reducing stomach pH-mediated breakdown. Prolonged use of PPI decreases the absorption of Vitamin B12 from the stomach, which leads to hyperhomocysteinemia and triggers endothelial dysfunction. Exposure of NO to superoxide free radicals amplifies oxidative stress in endothelial cells, contributing to endothelial dysfunction. These interconnected pathways collectively disrupt normal endothelial function, ultimately culminating in hypertension.

Endothelial dysfunction, marked by reduced availability of nitric oxide (NO), serves as a significant contributor to the risk of hypertension and cardiovascular disease and may represent a connection between the conditions. Studies indicate that NO has a crucial function in controlling blood pressure, and damaged NO bioactivity is an important factor in the development of hypertension [36]. The endothelium of blood vessels acts as a single cell layer located between the inner cavity of blood vessels and smooth muscle cells [37]. NO, a soluble gas, is consistently generated from the amino acid L-arginine within endothelial cells by an enzyme called nitric oxide synthase (NOS), which relies on calcium calmodulin for its activity [38]. There are three types of NOS found in endothelial cells: neuronal (nNOS), endothelial NOS (eNOS), and inducible NOS (iNOS). NOS requires various co-factors such as NADPH, tetrahydrobiopterin, oxygen, and flavin adenine nucleotides to function properly. The endothelial form of NOS is primarily responsible for the homeostasis of blood pressure [39]. In cases where endothelial dysfunction leads to a lack of NO release, agonists that normally cause blood vessel dilation instead trigger vasoconstriction by directly activating receptors in smooth muscle cells. This occurs due to increased intracellular calcium levels within the smooth muscle cells, which causes them to contract. However, when the endothelium is healthy, the potent vasodilatory effect of NO counteracts this response [40]. The guanidino-substituted analogs of L-arginine can block the formation of NO by acting competitively at the binding site of the enzyme. An example of such an analog is asymmetric dimethylarginine (ADMA), a substance detected in both human plasma and urine that functions as a natural antagonist of NO synthase. An increase in ADMA levels leads to endothelial dysfunction, which gains attention clinically due to the reduced ability of the endothelial to dilute blood vessels as well as elevated platelet aggregation and increased adhesion of monocytes [41]. ADMA is released during the breakdown of cellular proteins that possess methylarginine components, and its main removal occurs via the action of the enzyme dimethylarginine dimethylaminohydrolase (DDAH), a cytosolic enzyme ubiquitously expressed across various cell types. The primary reason for the increase in ADMA levels in the body is the disturbance in the DDAH function [42,43]. In a meta-analysis study, it was found that prolonged use of PPIs could potentially inhibit the activity of the DDAH enzyme and thereby decline the production of endothelial NO and impair vascular endothelial function [44]. In an ex vivo study, lansoprazole and omeprazole suppressed DDAH activity reversibly, resulting in a reduction in intracellular NO levels. This study concluded that impaired NOS activity in the cells could decrease the vasodilation function of the blood vessels, which could be responsible for the development of hypertension [45]. Another in vitro study explained that PPIs significantly increased the ADMA concentration via inhibition of the DDAH enzyme. PPI, when tested for concentration-dependent inhibition in the study, it was found that lansoprazole, omeprazole, and rabeprazole followed this pattern while esomeprazole and pantoprazole did not [46]. A cross-sectional analysis explained that the long-term use of PPI was evaluated in relation to the ADMA in the healthy population. This study showed evidence for the notion that PPI intake decreased NO availability in the endothelial cells and caused vascular endothelial dysregulation [44]. It has also been demonstrated that elevated plasma concentrations of ADMA can be seen in hypertensive patients [47]. Although endothelial dysfunction might additionally play a role in the elevated peripheral resistance by various pathways that result in increased constriction and structural, mechanical and functional changes in resistance arteries linked to the emergence and complexities of hypertension and its effects [48]. When NO production is compromised, as observed with PPI-induced endothelial dysfunction on prolonged use, there is a diminished ability to dilate blood vessels, contributing to increased vascular resistance and hypertension as shown in Figure 2. Esomeprazole, a PPI, was explored in an in vitro study using human microvascular endothelial cells. It negatively impacts the activity of the endothelial lysosomal enzymes and causes proteostasis which is involved in cellular dysfunction and acceleration of the aging process. This study explains how esomeprazole upregulated the expression of the p21 gene, a cell cycle inhibitor. In the esomeprazole-treated group, the expression of the plasminogen activator inhibitor (PAI-1) is upregulated which clearly indicates endothelial senescence. Furthermore, endothelial senescence is linked to the telomere length in endothelial cells. Six genes (POT1, TRF1, TIN2, TRF2, RAP1, and TPP1) encode the shelterin complex responsible for controlling and preserving telomere length and function, and results show that all six genes are downregulated by esomeprazole [49].

There is an alternate pathway for nitric oxide formation in the body, which comes from inorganic nitrate found in food and is quickly assimilated in the small intestine. This absorption leads to an increase in nitrate levels within the bloodstream, subsequently getting absorbed by the salivary glands. Nitrate is reduced to nitrite by nitrate reductase enzymes produced by commensal bacteria. When the saliva is exposed to the acidic environment of the stomach, nitrite undergoes a chemical reduction without the involvement of enzymes, resulting in the formation of NO and various other biologically active substances related to NO. Within this group of bioactive compounds, S-nitrosothiols function as stable donors of NO or reservoirs of NO and are linked to beneficial cardiovascular effects [50,51,52]. In one preclinical investigation, the impact of omeprazole and ranitidine was assessed in rats to determine the influence of stomach pH on the production of nitric oxide (NO) in the gastric region and bloodstream when provided prior to oral nitrates. The findings of the study provide compelling evidence to support the conclusion that the administration of stomach acid-reducing drugs, which result in an elevation in pH, effectively counteracts the hypotensive effects of oral nitrite treatment [53]. In a reported study, when rodents are treated with PPIs, such as omeprazole, it has been observed that the blood pressure-lowering effects of orally administered sodium nitrite are reduced. Oral sodium nitrite is typically used as a source of dietary nitrate and nitrite, which can be converted to NO in various tissues, including blood vessels. This conversion occurs via a process involving the reduction in nitrite to NO, mainly in an acidic environment such as the stomach. However, PPIs like omeprazole work by inhibiting the production of gastric acid, thereby increasing the gastric pH and creating a less acidic environment in the stomach. This change in pH can interfere with the conversion of nitrite to NO, leading to a reduction in the production of NO from dietary nitrate and nitrite. As a result, when rodents are treated with PPIs, the expected hypotensive effects of oral sodium nitrite are reduced. This affects the regulation of blood pressure in the body, which is typically mediated by the conversion of nitrite to NO, compromised due to the altered gastric environment caused by PPI administration [52,54,55]. In another investigation, the impact of omeprazole and ranitidine was assessed in rats to determine the influence of stomach pH on the production of nitric oxide (NO) in the gastric region and bloodstream when provided prior to oral nitrates. The findings of the study provide compelling evidence to support the conclusion that the administration of stomach acid-reducing drugs, which result in an elevation in pH, effectively counteracts the hypotensive effects of oral nitrite treatment. 

### 4.2. PPIs and Reduced Nitric Oxide Bioavailability via Vitamin B Deficiency

The absorption of vitamin B12 is primarily dependent on the intact gastric corpus mucosa, which includes parietal cells found in the gastric glands of the stomach corpus region. These cells have a crucial role in producing gastric acid and intrinsic factors, both necessary for efficient vitamin B12 absorption [56]. A study conducted to investigate the relationship between extended utilization of PPI and H2RA (H2 receptor antagonist) use and the occurrence of vitamin B12 deficiency revealed a significant association. The findings suggest that the prolonged usage of these acid-releasing inhibitors may increase the likelihood of developing a deficiency of vitamin B12 [57]. A recent cross-sectional study revealed a significant correlation between elevated plasma homocysteine levels and the occurrence of hypertension, taking into account plasma folate and vitamin B12 levels [58]. Increased concentration of homocysteine in the blood is the most common laboratory marker commonly used to indicate this deficiency. These increased homocysteine levels have also been found to have a significant association with vascular deficits [59,60,61].

Vitamin B12 is essential for transforming homocysteine into methionine, and its deficiency results in hyperhomocysteinemia, which directly relates to hypertension [58,62]. Homocysteine is a byproduct of methionine metabolism, and high levels of homocysteine in the blood, known as hyperhomocysteinemia, have been linked to a range of negative impacts on the cardiovascular system [63,64].

The elevated homocysteine levels can impact the cardiovascular system by impairing the vasodilation process mediated by reducing the availability of NO via an enzyme asymmetric dimethylarginine (ADMA) by inhibition of NO synthetase [65]. NO serves as a strong vasodilator, inducing the relaxation and expansion of blood vessels to support optimal circulation of blood. However, homocysteine can interfere with the bioavailability and action of NO, leading to reduced vasodilation and impaired blood vessel function [66]. Furthermore, elevated homocysteine levels can increase oxidative stress within the blood vessels. Excessive homocysteine can lead to increased ROS production, which can damage the endothelial cells that coat the inner walls of blood vessels and promote inflammation and atherosclerosis [67,68].

Homocysteine also triggers the growth of smooth muscle cells found in the middle layer of the blood vessel walls. Excessive growth of these cells can contribute to the formation of atherosclerotic plaques, leading to narrowing the blood vessels and compromising blood flow [69]. Moreover, elevated homocysteine levels can alter the elastic properties of the vascular wall. This can result in increased arterial stiffness, which is associated with higher blood pressure [70]. In summary, elevated homocysteine levels can negatively affect the cardiovascular system by impairing vasodilation, increasing oxidative stress, stimulating smooth muscle cell growth, and altering the elasticity of blood vessels, leading to the progression of hypertension, as depicted in Figure 2 [70,71]. 

### 4.3. PPIs Potentially Enhance Vasoconstriction through Hypocalcaemia

Calcium compounds present in the food need to undergo ionization before calcium ions with a positive charge can be taken in the duodenum or small intestine with increased ionization and solubility in an acidic environment. The pH level significantly impacts the solubility of calcium, with the secretion of gastric acid and gastric acidity playing a crucial role in the absorption of calcium from less soluble forms like calcium carbonate and dietary sources. Currently, there is limited evidence from both animal and human research indicating that chronic use of PPI might lead to a decrease in the absorption of insoluble calcium [72]. It is believed that calcium is primarily absorbed in its ionized state in the upper small intestine, and this ionization is aided by an acidic environment to free calcium from its salt form or food complex. Preclinical research concludes that calcium absorption could be diminished by PPIs, H2RAs, and achlorhydria, while acidic conditions might enhance calcium uptake [73,74]. Insufficient acid secretion can result in minimal dissolution (ionization) of calcium salts, leading to inadequate and less efficient absorption of calcium [75]. In a study, omeprazole was administered with the diet to evaluate its effect on calcium absorption from the small intestine, and it found that significant suppression of gastric acid secretion increases the pH in the stomach and affects intestinal calcium absorption [75,76]. PPIs hinder both natural and stimulated secretion of gastric acid, resulting in elevated gastric pH levels that could potentially impact the ionization and uptake of calcium compounds. In an animal study, it was demonstrated that pantoprazole may cause retardation of calcium absorption by inhibiting the gastric proton pump [77]. 

In a rabbit model, the impact of omeprazole and esomeprazole on calcium levels was investigated over a period of three months. The findings revealed a remarkable reduction in serum calcium levels in the omeprazole group, whereas no connection was proposed between decreased serum calcium levels and the use of esomeprazole in the study [78]. O’Connell et al. conducted a randomized crossover trial involving menopausal women who received 20 mg of omeprazole for seven days, followed by a calcium supplement. The level of calcium was measured 5 h after ingestion without food during the interval. The findings indicated that omeprazole therapy had a significant impact on reducing calcium absorption from calcium carbonate when taken by older women on an empty stomach after an overnight fast [79]. Graziani et al. conducted a different study where it was observed that omeprazole led to a reduction in calcium plasma levels when administered to healthy subjects prior to eating [80]. The exact mechanism through which PPIs affect bone metabolism remains uncertain. One hypothesis proposes that PPIs could potentially decrease the absorption of calcium in the intestines by raising the gastric pH, which may, in turn, impact the dissolution of calcium salts derived from dietary sources. A consequence of reduced calcium absorption is an elevated risk of bone breakdown and secondary hyperparathyroidism, ultimately resulting in an unfavorable calcium imbalance [31,81]. Clinical studies have reported a clear association between inadequate calcium consumption and elevated levels of parathyroid hormone (PTH) levels, which can lead to hypertension in the long term. Hence, these studies showed an indirect correlation between calcium levels and the blood pressure of an individual [82,83,84,85]. Low calcium intake can have a direct impact on the synthesis of calcitriol or be mediated by parathyroid hormone (PTH), both of which contribute to increased calcitriol production. Additionally, low calcium intake stimulates the release of renin, which leads to the synthesis of angiotensin II. The combined effects of these mechanisms ultimately result in hypertension caused by insufficient levels of calcium [86,87]. 

### 4.4. PPI-Induced Hypomagnesemia and Damage to Vascular Function

Hypomagnesemia, which refers to abnormally low levels of magnesium in the blood, recently gained recognition as a potent side effect of PPI therapy before the FDA issued a warning in 2011 [88]. Following the FDA’s warning, several clinical studies were undertaken to investigate the potential association between chronic PPI use and magnesium deficiency. These studies consistently demonstrated a significant link between chronic PPI treatment and reduced magnesium levels among patients [89,90]. Magnesium deficiency can lead to various adverse effects on the body, as magnesium is an essential mineral involved in numerous physiological processes. However, the precise mechanism by which PPIs contribute to magnesium deficiency is not fully understood [91]. It has been believed that PPIs may obstruct the absorption of magnesium in the intestines, leading to reduced magnesium levels over time and disrupting the balance of magnesium homeostasis in the body [92]. 

The consequences of magnesium deficiency can be significant as magnesium plays a vital role in various activities, such as controlling blood pressure, strengthening the immune system, sustaining proper muscle and nerve conduction, and promoting bone health. Therefore, chronic magnesium deficiency resulting from extended PPI use may lead to a range of symptoms and complications, including muscle weakness, tremors, cardiac arrhythmias, fatigue, and osteoporosis [93]. 

**Figure 3:**
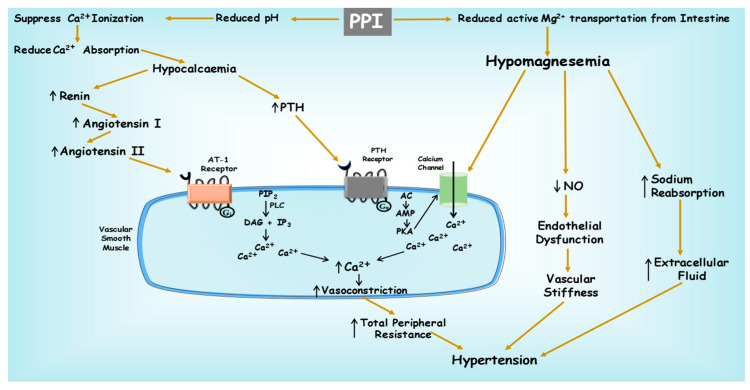
PPIs are linked to the inhibition of calcium and magnesium absorption from the gastrointestinal tract (GIT) into the bloodstream, leading to hypocalcemia and hypomagnesemia. These deficiencies in minerals within the bloodstream trigger an elevation in intracellular calcium levels through the activation of the AT-1 and PTH receptors. Consequently, there’s an augmentation in calcium influx within vascular smooth muscles, due to which constriction of blood vessels increases. This process results in an increase in overall peripheral resistance and subsequently leads to hypertension. Additionally, hypomagnesemia fosters enhanced sodium absorption, which in turn elevates extracellular fluid levels and contributes to hypertension.

Both clinical and pre-studies have indicated the association between magnesium (Mg2+) deficiency and the development or worsening of hypertension. Clinical studies have demonstrated a consistent link between low magnesium levels and the incidence of hypertension. Research has shown some evidence that individuals with lower magnesium intake or lower serum magnesium levels are at a higher risk of developing hypertension. Furthermore, long-term observational studies have indicated that individuals with magnesium deficiency have an increased likelihood of experiencing an increase in blood pressure over time, as shown in Figure 3 [94,95].

The connection between low magnesium levels and high blood pressure is complex. Magnesium is crucial for regulating vascular tone, endothelial function, and the balance of electrolytes such as calcium and potassium. In magnesium deficiency, disturbances in these mechanisms can occur, leading to vasoconstriction, impaired endothelial function, and altered sodium-potassium balance, which can subsequently contribute to high blood pressure [96,97,98].

## 5. Conclusions and Future Perspective

Hypertension is a prevalent medical condition that can lead to serious and life-threatening consequences, including stroke, kidney failure, vision impairment, and various cardiovascular diseases. Its multifactorial etiology encompasses a complex interplay of genetic predisposition and lifestyle factors, which makes it challenging to precisely pinpoint the singular cause of hypertension [99]. Evidence shows that irrational use of medications can potentially lead to the development of hypertension as an unintended side effect [100]. PPIs are routinely recommended drugs for the relief of acid-related illnesses such as peptic ulcers and GERD. While these drugs are typically regarded as safe and effective, studies reveal a potential connection between long-term PPI usage and a risk of hypertension [1]. However, new studies have generated curiosity about the possible effects of chronic PPI usage on blood pressure. Various mechanisms are responsible for the homeostasis of the blood pressure altered by the abuse of PPI. Chronic use of PPIs inhibits the DDAH enzyme in the endothelial cells, which is responsible for ADMA metabolism. Increased ADMA directly blocks the formation of NO, which is responsible for endothelial dysfunction. PPI also reduces the absorption of vitamin B12, resulting in endothelial dysfunction by a similar pathway. Additionally, PPI causes hypocalcemia and hypomagnesemia, which increase intracellular calcium and lead to vasoconstriction. All these mechanisms elevate the total peripheral resistance and ultimately lead to hypertension. Since the etiology of hypertension is multifactorial, these mechanisms can contribute to the dysregulation of blood pressure. The question of whether prolonged PPI use is connected to an increased risk of hypertension is a topic that continues to be explored. While the relationship between PPIs and hypertension requires further exploration, the evidence suggests the need for cautious prescribing and a balanced approach to PPI usage. Understanding these pathways provides valuable insights into the complex interplay between PPIs and hypertension, enabling better patient management and the development of strategies to mitigate this possible side effect. However, present clinical and preclinical evidence are not enough to prove the exact correlation between chronic PPI use and hypertension. As additional research progresses, a better understanding of the connection between PPIS and hypertension will emerge. Further research is needed to establish a definitive causal relationship and develop guidelines for healthcare providers. Awareness among clinicians and regular blood pressure monitoring in patients on long-term PPI therapy is crucial to ensuring optimal management of their overall health.

## Figures and Tables

**Table 1:** Shifting Patterns in PPI Prescription Trends around the world in the 21st century.

Study Type	Population	Country	Time Period	Inference	Reference
Cohort analysis	Population of age more than 18 years	UK	1990–2014	PPI prevalence increased up to 15%, in which 26% used PPI long term and 3.9% used it for 5 years.	[18]
Retrospective observational study	Infants of age less than 1 year	United States of America	1999–2004	PPI usage increases four times in infants; lansoprazole and omeprazole are used mainly.	[19]
Prescription analysis	Adults aged between 5 and 15 with a median age 12	Denmark	2000–2014	PPI use raised to 4 times	[20]
Prescription analysis	Children of Denmark aged between 0–17 years	Denmark	2000–2015	A total of 8 times increment in the dispensing rate of PPI in Danish children’s prescriptions, and omeprazole is the most frequently used PPI	[21]
Retrospective analysis	Patients of the emergency department aged more than 65	United States of America	2001–2010	Increase in prescription rate from 3% to 7.2%, with pantoprazole being the most utilized.	[22]
Prescription analysis	Individuals of any age group with a mean age 62	Spain	2002–2015	Increase in the global dispensing of PPIs from 12.5% in 2002 to 18.1% in 2015. Omeprazole is the most prescribed PPI in this period, and the most frequent age group was above 65	[23]
Retrospective study	Population of age between 18 and 80	China	2007–2016	Prescription rate in inpatient increased from 20.41% to 37.21%.	[24]
Retrospective study	Population of age 18 or older with mean age 51.2	Switzerland	2012–2017	Inappropriate PPI prescription increased from 4.8% to 6.4%, and the annual incidence of PPI also rose to 4%	[25]
Cross-sectional retrospective study	Adult population of age 30–40 years	Saudi Arabia	2019	Increase in the use of PPI, with pantoprazole being the most prescribed	[26]

## Data Availability

Not applicable.
